# Red Blood Cell Deformability in Microfluidic Constrictions Under Flow and Wall Contact

**DOI:** 10.3390/mi17060670

**Published:** 2026-05-28

**Authors:** Keigo Nonomura, Mitsuhiro Horade, Yuta Shirasaka, Shuichi Murakami, Hiroaki Ito, Kenichiro Yoshitomi

**Affiliations:** 1 Department of Mechanical Engineering, National Defense Academy of Japan, 1-10-20 Hashirimizu, Yokosuka-shi 239-8686, Kanagawa, Japanyokken@nda.ac.jp (K.Y.); 2 Department of Mechanical Engineering, Setsunan University, 17-8 Ikedanaka-cho, Neyagawa-shi 572-8508, Osaka, Japan; 3 Department of Electronic and Mechanical Systems, Osaka Research Institute of Industrial Science and Technology, 2-7-1 Ayumino, Izumi-shi 594-1157, Osaka, Japan; sh-murakami@orist.jp; 4 Graduate School of Science, Chiba University, 1-33 Yayoi-cho, Inage-ku, Chiba-shi 263-8522, Chiba, Japan; ito@chiba-u.jp

**Keywords:** micro-fluidic device, capillary, micro-pump, cell behaviour, red blood cells (RBC)

## Abstract

Microfluidic devices are widely used for cell manipulation, but the effects of physical contact between cells and microchannel walls are not well understood. This study examines how such contact influences the behaviour of red blood cells (RBCs) during controlled manipulation. RBCs were driven through a narrow microchannel constriction (3.6 × 3.0 µm in cross-section and 2500 µm in length), enabling precise application of mechanical load. A pump system allowed accurate control of flow conditions, ranging from complete immobilisation to defined shear stress by adjusting flow rates. Under immobilised conditions, the recovery time constant of RBCs increased with longer loading durations, consistent with previous studies. However, when shear stress was introduced, recovery dynamics changed significantly. Notably, a 30-fold difference in recovery time constant was observed between a 5 s immobilisation and a 5 s load applied at a flow speed of 0.5 mm/s. Furthermore, the rapid elastic recovery typically occurring within approximately 0.1 s after unloading was suppressed under flow conditions. These results demonstrate that viscous interactions between channel walls and surrounding fluid play a critical role in determining cellular responses during microfluidic manipulation.

## 1. Introduction

This study investigates cellular behaviour utilising a microfluidic device designed to apply a controlled load to red blood cells (RBCs) and assess their recovery post-unloading. Numerous studies have reported on the measurement of cell characteristics using micromanipulation technologies. These prior works can be broadly classified into three categories: precision manipulation, cell characteristic measurement, and RBC recovery characteristics. Under the first category, various methods have been developed for manipulating objects at the micron scale. One notable technique involves the use of optical tweezers, which offer the advantage of non-contact manipulation. Minute forces are employed to move objects, thereby minimising potential damage to cells [1,2]. The underlying principles of this technique have been elucidated [1], and advancements aimed at reducing irradiation time have also been reported [2]. Methods in which glass tubes are processed into needles or pipettes and utilised as end effectors for the physical transport of robotic arms have also been reported [3,4,5,6]. Beyond basic grasping operations, innovative applications have emerged that integrate virtual reality [3] and advanced control mechanisms for end-effector movement [4]. Additionally, methods akin to non-contact operations using magnets have been developed [5], with practical applications, such as drug administration [6]. The robot arm approach offers the distinct advantage of three-dimensional manipulation, whereas microfluidic devices are recognised for their high throughput capabilities. Various methods have been proposed for cell manipulation and positioning, including the use of dielectric forces [7], surface acoustic waves [8], and physical supplementation [9]. Additionally, advancements in size-based cell sorting have emerged through various approaches, including the use of unique flow patterns [10], centrifugal forces [11], and channel shape optimisation for filter structures [12]. In the second category of research, which focuses on measuring cellular characteristics using microfluidic devices, studies have adopted image-based methodologies to assess cell stiffness and deformation [13,14,15,16], owing to the challenges of integrating micro-force sensors. The target cells in these studies are diverse, encompassing RBCs [13], neutrophils [14], NIH 3T3 fibroblast cells [15], HeLa cells [16], and cancer cell selection [17]. Furthermore, techniques involved in measuring chemical properties exist [18,19,20,21], which are instrumental in analysing proteins [18], metabolites [19], and miRNAs [20]. Various approaches have been employed, including simulation-based research [21], electrical measurement methods [22], and sorting based on deformability [23,24]. Research dedicated to the shape recovery characteristics of RBCs has utilised a range of techniques, such as micropipette aspiration [25], optical tweezers [26], atomic force microscopy [27,28], flicker spectroscopy [29], and microfluidic devices [30]. Given the operational complexities associated with robot arm-type manipulators, which require a high level of skill to operate effectively, this study leveraged a microfluidic device known for its high throughput capabilities. Among the research groups that use microfluidic devices to evaluate the viscoelasticity of minute objects, there are many reports evaluating the viscoelasticity of hydrogels. These studies focus on sodium alginate [31], xanthan gum [32], agarose [33], etc., due to their direct relevance to applications such as cell encapsulation and drug release. This has been evaluated both experimentally and numerically [34,35]. Many reports involve measuring mechanical viscoelasticity by changing the gel formulation conditions and ultimately utilising this information for accurate droplet generation technology. In droplet generation research, the target size is large, on the order of tens of micrometres, but parameter evaluation is often applied to microfluidic devices. On the other hand, with regard to RBCs, there are more reports that evaluate only the cell membrane compared to examples that evaluate the viscoelasticity of the entire object, such as hydrogels. In this study, we focused on the viscoelasticity of the entire object, which is similar to a hydrogel, but when considering why the viscoelasticity changes, it is necessary to consider the cell membrane and structure as a basis.

Flicker spectroscopy and atomic force microscopy are effective techniques for assessing cell membranes [27,28,29]. The viscoelasticity of whole cells can be evaluated using a microfluidic device; these viscoelastic parameters vary depending on the presence or absence of adenosine triphosphate [30]. In a previous study, RBCs were modelled as viscoelastic bodies using a standard linear elasticity (SLE) framework, with a particular emphasis on their constituent components. For normal RBCs, the damping components within the parallel circuit significantly influence the recovery time constant following a 180 s load application, resulting in a prolonged recovery time. Alternative methodologies for assessing the deformability of RBC membranes, such as the Skalak model [36] and Evans model [37], have also been reported. However, these models lack a viscous component, which limits their ability to accurately replicate time-dependent behaviours.

In this study, we aim to investigate the viscoelasticity of the entire RBCs rather than focusing solely on the cell membrane. For example, if the purpose is to evaluate only the elastic component, only the behaviour within a microchannel can be assessed [38]. However, because our focus is on shape recovery behaviour, we will design a microfluidic device that facilitates the observation of RBCs post-load application owing to constriction. Furthermore, as previously mentioned, existing research has utilised microfluidic devices to measure the shape recovery behaviour of RBCs after they have been confined in a constricted area for a specified duration and subjected to compressive loads. In a previous study, a pump was utilised that integrated a high-speed camera and piezoelectric actuator to precisely control the position of the pump, enabling it to remain at the constriction for a specified duration [30]. The channel length in that study was 10 µm, which limited the ability to conduct load application experiments under flow conditions. Consequently, the objective of the current study was to perform load experiments in a long channel using a pump that maintained a constant flow rate over an extended period.

## 2. Materials and Methods

### 2.1. Design Guidelines

**Figure 1:**
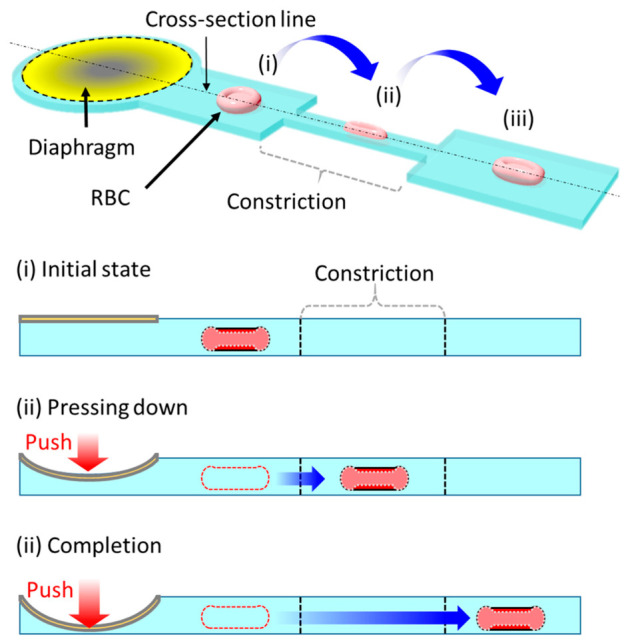
Conceptual diagram of RBC manipulation utilising a diaphragm pump. Bird’s-eye view and cross-sectional line, as well as the image of cell manipulation on the cross-sectional line. A thin film of highly deformable PDMS was used as the diaphragm. When the diaphragm was pressed, the volume of the flow channel changed, resulting in the movement of the object being manipulated. (**i**) In the initial state, the focus was on the RBC located between the constriction and the diaphragm. (**ii**) When the diaphragm was pressed down, RBCs entered the constriction and a load was applied. (**iii**) With further pressing, the RBCs were released from the constriction, enabling the observation of their recovery behaviour once the load was removed. By controlling the amount of pressure applied, we could facilitate both reciprocating motion and the ability to halt movement within the constriction.

In this study, we introduced a constriction that simulated a capillary with a cross-section of 3.6 × 3.0 µm and a length of 2500 µm, combined with manipulation technology, to apply a load to RBCs. The length of capillaries in the body was approximately 0.6 mm [39]. However, diseased RBCs are known to exhibit a unique recovery response when subjected to stress for 180 s or longer [30]. With future applications in diagnostics and disease-state evaluation using RBCs in mind, the channel length was set to 2500 µm to enable the application of shear stress for 180 s or longer. For liquid delivery and manipulation, we implemented a method that integrated a polydimethylsiloxane (PDMS) diaphragm pump within the microfluidic device. This study required operations such as low-speed movement, stopping within the channel, and reverse motion. These operations are difficult to achieve using commercially available syringe pumps equipped with stepping motors. Even when the pump is stopped remotely via tubing, the object within the channel does not stop immediately because of tubing deformation and the resulting delay [40]. The design concept is shown in Figure 1. Specifically, we focused on the integration of a diaphragm structure within a specific section of the device that was directly connected to the main channel. Applying pressure to the diaphragm caused the RBCs, which were the target objects for manipulation, to be directed toward the constriction (Figure 1). The deformation of the diaphragm was adjusted as an input value to manipulate the object by pre-determining the degree of movement and speed of the object based on the degree of depression. Moreover, the diaphragm’s ability to return to its original position was facilitated by the elastic restoring force of PDMS, which was the material utilised in the device’s construction. This characteristic enabled seamless integration of the pump by simply substituting a portion of the device with a diaphragm-shaped component. Previous studies have reported the successful application of this technique [41,42], and we adopted it in our research owing to its capacity to incorporate a small pump while preserving the constriction structure, thereby enabling fine control over a minute flow rate. Additionally, the diaphragm’s significant deformation capabilities, surpassing those of silicon diaphragms, have led to its use as a pressure sensor [43]. In this study, a PDMS thin-film diaphragm was directly deformed using an indenter to drive the pump. Furthermore, by attaching a micrometre to the indenter, we could precisely set the indentation depth to 1 µm. Moreover, by incorporating a piezoelectric element, the indentation depth could be set with a minimum resolution of 1 nm. In our manipulation of RBCs, we installed a piezo and calibrated the pushing force to ensure consistent operational speed. This approach enabled us to maintain the advantages of the inexpensive and highly versatile PDMS microfluidic device, facilitating the replacement of the device for each cell experiment without compromising its advantages.

**Figure 2:**
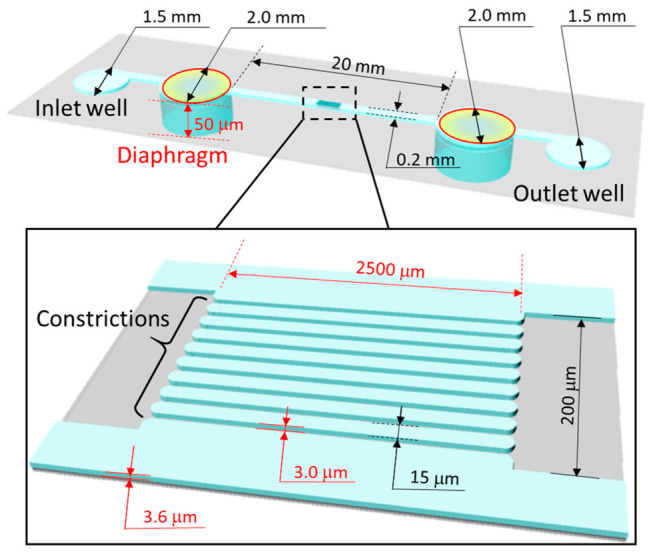
Dimensions of the microfluidic device. The device comprised an inlet well, an outlet well, diaphragms, and constrictions. Owing to its linear symmetrical design, the inlet well and outlet well could be interchanged; however, only the diaphragm closest to the inlet well was actuated in this experiment. The channel between the diaphragms was linear, with a length and a width of 20 mm and 200 µm, respectively. An array of nine constrictions, with a width of 3 µm and a length of 2500 µm, was positioned at the centre of this channel. The diaphragm section had a depth of 50 µm, while the remaining channels were 3.6 µm deep.

The microfluidic device was designed with inlet and outlet wells to introduce and discharge the cell suspension, respectively; it also included a diaphragm and a constriction. A schematic of the device and its design dimensions is shown in Figure 2. Both the inlet and outlet wells had a diameter of 1.5 mm, whereas the two diaphragms each measured 2 mm in diameter. Owing to the axisymmetric structure, the inlet and outlet wells were interchangeable; however, only the diaphragm adjacent to the inlet well was actuated in this experiment. The channel between the diaphragms was linear, with a length and width of 20 mm and 200 µm, respectively. An array of nine constrictions, each with a width of 3 µm and a length of 2500 µm, was positioned at the centre of this channel. In the case of a single constriction, the reduction in the channel width from 200 µm to 3 µm resulted in an approximately 66-fold increase in the migration speed of RBCs at the constriction compared with the speed outside the constriction, complicating the control of RBC speed. In contrast, when nine constrictions were employed in an array, the speed was reduced to approximately 7.4 times, facilitating better control over the movement of the cells. The tilting of the RBCs in the vertical direction was suppressed by setting the channel depth to 3.6 µm, a value validated by measurements utilising a white light interferometer (NewView6300; Zygo Corporation, Middlefield, CT, USA) on the experimental device. However, the depth of the diaphragm section was set to 50 µm. The diaphragm diameter was set to 2 mm, considering the pump capacity necessary for effectively manipulating RBCs over a distance exceeding the length of the constriction, as well as the ease of positioning the diaphragm when actuated by an indenter. Preliminary experiments demonstrated a linear relationship between the degree of diaphragm depression and the corresponding movement of RBCs, with a maximum depression threshold of 100 µm. For example, a diaphragm depression of 100 µm resulted in an RBC displacement of 12.3 mm. Consequently, maintaining a diaphragm depth of 50 µm provided adequate manoeuvrability for the RBCs to navigate through constrictions measuring up to 2500 µm.

### 2.2. Design Guidelines of the Microfluidic Device

**Figure 3:**
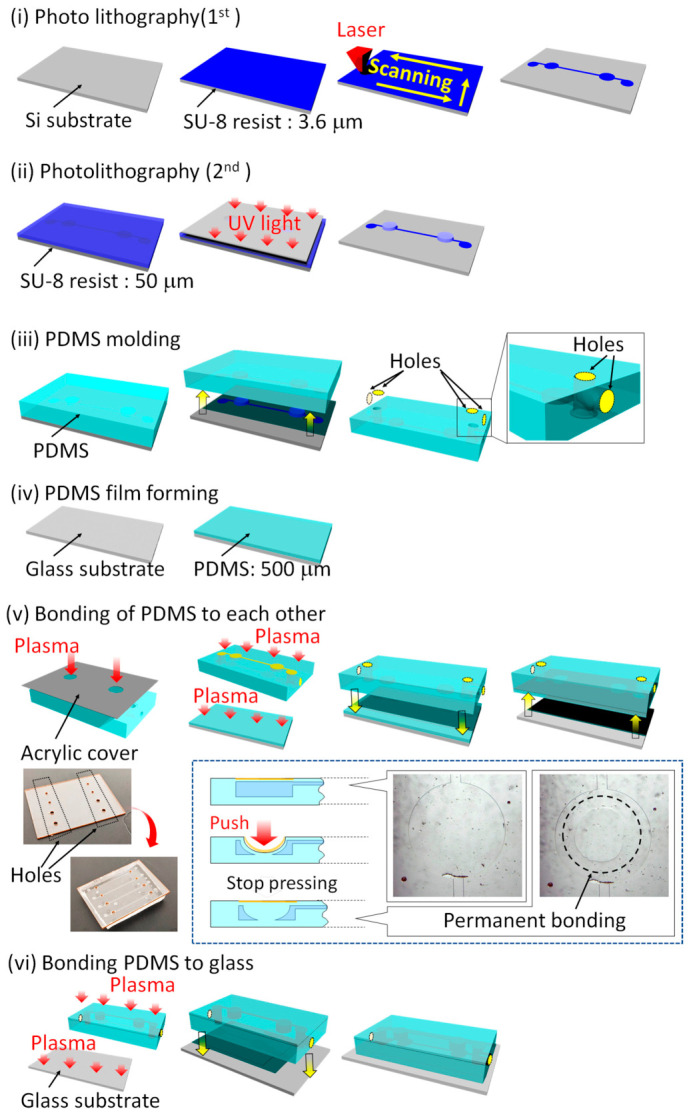
Fabrication of the microfluidic device.

The next phase of the process involved fabrication (Figure 3). (i) The initial step was photolithography, where “SU-8 3005” resist (KAYAKU Advanced Materials, Inc., Tokyo, Japan) was applied onto a silicon substrate at 7000 rpm. A soft bake was conducted at 95 °C for 5 min using a hot plate to obtain a substrate of a thickness of 3.6 µm. Subsequently, the channel pattern was exposed using a maskless exposure device (MLA150; Heidelberg Instruments, Heidelberg, Germany). Following a post-exposure bake at 95 °C for an additional 5 min, the substrate was developed in PGMEA (propylene glycol monomethyl ether 1-methoxyl-2-propanol) for 5 min. (ii) The second photolithography step was then performed. “SU-8 3050” resist (KAYAKU Advanced Materials, Inc., Tokyo, Japan) was applied at 3000 rpm. A soft bake at 95 °C for 30 min was performed using a hot plate to obtain a substrate of a thickness of 50 µm. The cells were exposed to UV light at 250 mJ/cm^2^ using a mask aligner (MA-6; SUSS MicroTec, Munich, Germany). After a post-exposure bake at 95 °C for 7 min, the film was developed in PGMEA for 15 min. (iii) After film development, PDMS (Silpot 184; DuPont Toray Specialty Materials K.K., Tokyo, Japan) was poured onto the substrate to a thickness of approximately 10 mm. After vacuum degassing for 30 min, the samples were cured in an oven at 80 °C for 30 min. Once cured, the PDMS was carefully released from the board, and a 1.5 mm punch was utilised to create holes in the inlet and outlet wells. (iv) For the formation of the thin PDMS film constituting the diaphragm, vacuum-degassed liquid PDMS was applied to a glass substrate at 100 rpm, resulting in a film thickness of approximately 500 µm. This thickness was determined based on preliminary experiments, which considered both the deflection caused by the diaphragm’s weight and its ability to return to its original shape. The mixture was then baked on a hot plate at 95 °C for 10 min for hardening.

(v) The channel structure was then bonded to the thin film through the application of air plasma using a hydrophilic device (PIB-20; Shinku Device Inc., Ibaraki, Japan). A key design strategy was to mask a portion of the PDMS surface to prevent adhesion. Optimal adhesion occurred at a specific dosage of plasma exposure; however, adhesion deteriorated when an excessive dosage was applied. Preliminary experiments revealed that adhesion was successful at an exposure dosage of 186.6 J/cm^2^, whereas no adhesion was observed at 334.2 J/cm^2^. Furthermore, when the diaphragm was pressed down to a greater depth, contact between the thin film of the diaphragm and the underlying surface resulted in a permanent bond that could not be peeled off. Figure 3v shows a schematic diagram and a microscopic image of permanent bonding achieved by pressing. This unintended adhesion compromised the diaphragm’s structural advantage of returning to its original shape. To mitigate this issue, we increased the amount of plasma exposure to prevent premature adhesion. Furthermore, the depth of the diaphragm was set to 50 µm, reducing the likelihood of adhesion even when the diaphragm was bent. First, an acrylic cover was employed to deliver an exposure dosage of 334.2 J/cm^2^ to the diaphragm. Following this, the acrylic cover was removed, and the adhesive surfaces were subjected to an exposure dosage of 186.6 J/cm^2^. (vi) The thin films on the glass substrate and channel structure were bonded and baked at 95 °C for 10 min using a hot plate. Upon peeling the PDMS from the glass substrate, both the thin film and the bulk material could be removed while maintaining their bonded state. After bonding, the backside of the PDMS and the glass substrate were exposed to air plasma at 186.6 mJ/cm^2^. The PDMS and glass substrates were then bonded together and baked at 95 °C for an additional 10 min on a hot plate to finalise the procedure.

### 2.3. Experimental Method

**Figure 4:**
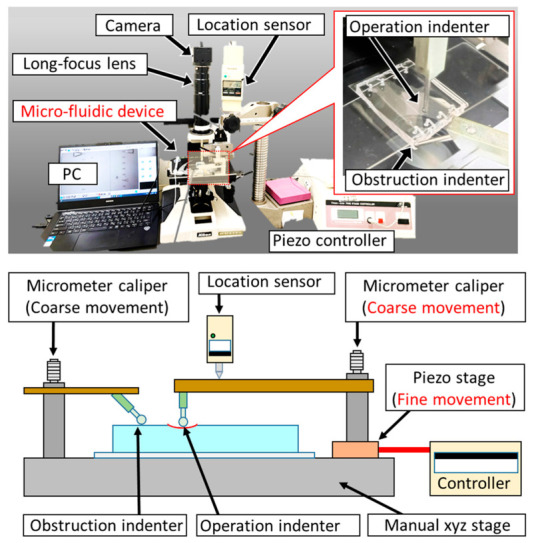
External view and schematic of the experimental equipment. The experimental setup comprised several key components: a microfluidic device, long-focus lens, camera, and two indenters. One indenter was designated for driving the diaphragm, whereas the other served to seal the inlet well. The diaphragm-driving indenter was equipped with a position sensor and piezoelectric stage. Coarse adjustments to the indenter were performed using a micrometre, whereas fine adjustments were achieved through the piezo stage.

The external appearance and schematic of the experimental equipment are shown in Figure 4. The primary components include a microfluidic device, long-focus lens, camera, and two indenters. To prevent physical interference with the indenter used to actuate the diaphragm, we used a long-focus lens (VSZ-M07545VS; VS Technology Corporation, Tokyo, Japan) with a maximum magnification of 22.5× and a focal length of 95.25 mm in the microscope. A camera (BU1203MCF; Toshiba Teli Corporation, Tokyo, Japan) captured images at 22 fps with a resolution of 2448 × 1882 pixels. The maximum image resolution used in this case was 0.13 µm/pixel. The selected imaging parameters were carefully determined based on several factors, including the loading time and writing speed necessary for recording the recovery behaviour, spatiotemporal resolution required for observing cell behaviour, and preliminary experiments. In conjunction with the microfluidic device, a manual three-axis stage (XYZ axes) was integrated. An indenter was designed to actuate the diaphragm and equipped with a micrometre for coarse adjustments; a position sensor, piezo stage, and controller were incorporated for fine adjustment. The second indenter was employed to apply pressure to the inlet well, effectively sealing the channel, and was similarly outfitted with a micrometre for coarse adjustments. The cell suspension was prevented from flowing back into the inlet well during diaphragm actuation with a movement resolution on the order of submillimetres. The micrometres attached to both indenters featured a scale of 10 µm and were operated manually. The position sensor (U60A; SONY GROUP CORPORATION, Tokyo, Japan) offered a minimum resolution of 1 µm, enabling precise measurement of the coarse adjustment micrometre’s depression. Additionally, the piezoelectric stage (SFS-H60XYZ(CL); SIGMAKOKI CO., LTD., Saitama, Japan) had a minimum movement resolution of 1 nm and repeatable positioning accuracy of 0.2 µm.

**Figure 5:**
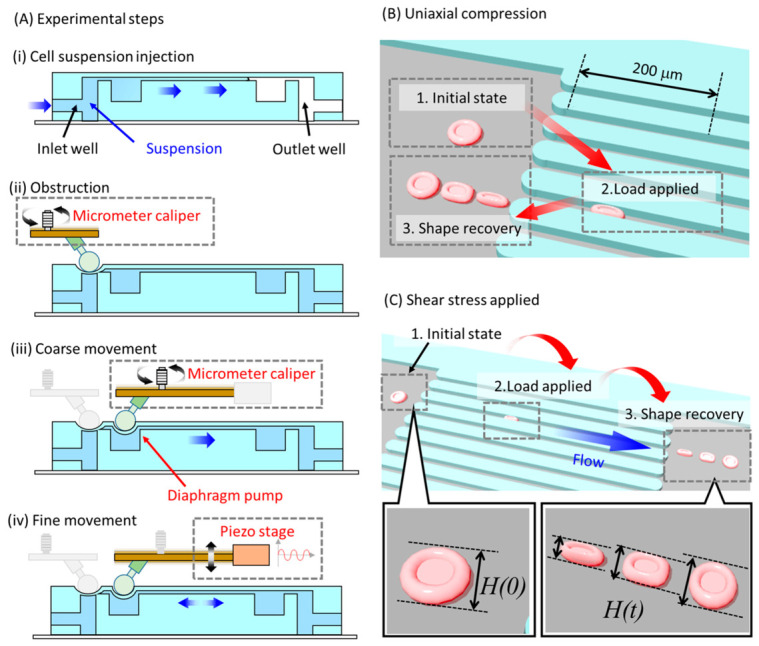
Experimental method. (**A**) Overview of the operation of the experimental equipment. (**i**) Injection of cell suspension. (**ii**) Closure of the inlet well using an indenter. (**iii**) Alignment of the indenter with the diaphragm through coarse movement operation. (**iv**) Precise operation of the diaphragm by operating the piezo stage. (**B**) Experimental method in which RBCs were immobilised within a channel while a uniaxial compression load was applied. (**C**) Experiment demonstrating the application of shear stress while RBCs flowed through a channel.

The experimental procedure is shown in Figure 5A. The steps were as follows: (i) Cell suspension injection: A cell suspension was introduced into the device through the inlet well. The cell suspension was prepared by diluting the blood sample approximately 1000 times with phosphate-buffered saline. Blood was taken with informed consent from healthy voluntary donors. Blood withdrawal, sample preparation, and experiments were performed according to regulations and protocols that were approved by the ethics committee of the “Setsunan University Ethical Review Committee for Medical and Health Research Involving Human Subjects” (reference no. 2025-034). Dilution reduced the hematocrit value to 0.05% or less. The dilution was to prevent multiple RBCs from entering the constriction. (ii) Inlet channel blocking: To prevent backflow of liquid from the pump to the inlet side—an occurrence that could compromise or destabilise cell movement—the inlet channel was occluded using a spherical indenter with a diameter of 2 mm. (iii) Diaphragm operation: The diaphragm was actuated by pressing it down with the indenter. Prior to this step, the pressing position was adjusted using the coarse adjustment micrometre. For applications requiring positioning accuracy within a single-digit micron range, height adjustments were performed solely through the position sensor and coarse adjustment stage. (iv) Fine adjustment: In scenarios demanding even greater precision, the piezoelectric stage was employed for fine adjustments. Two types of RBC loading experiments were conducted. The first experiment focused on immobilising RBCs within a channel and applying a load to them, a technique referred to as uniaxial compression (Figure 5B). Initially, the RBCs were introduced into the constriction, where they were held stationary for a predetermined duration. Finally, the diaphragm was retracted to release the RBCs from the entrance of the constriction, enabling the observation of their shape recovery behaviour. To minimise the impact of shear stress on the RBCs during both their introduction and release from the constriction, we set the stopping position of RBCs at the constriction within 200 µm from the entrance of the constriction. The loading times were established at *T* = 5, 60, 180, and 300 s, consistent with previous studies [30]. Since the channel width was 3 µm, the applied load was estimated to be approximately 25 pN. An RBC with a diameter of 8 µm was compressed by 5 µm, and the calculation was based on an RBC spring constant of 5.0 × 10^−6^ N/m [44]. The contact length between the channel wall and the RBC was approximately 10 µm. Therefore, the compressive stress was estimated to be approximately 1.25 Pa. The second experiment involved the application of shear stress while RBCs flowed through a channel (Figure 5C). In this setup, RBCs were introduced into the constriction and subsequently propelled through it at a constant flow rate. Upon exiting the constriction, the shape recovery behaviour of the RBCs was again observed. Given that the constriction measured 2500 µm in length and extended beyond the microscope’s field of view, the stage of the microfluidic device was manually adjusted to ensure that the RBCs remained within the field of view of the microscope. The loading times were *T* = 5, 60, 180, and 300 s. To facilitate the passage of RBCs through the 2500 µm constriction within the specified loading times, their velocities were adjusted to 0.5, 0.042, 0.014, and 0.008 mm/s, respectively. The corresponding shear stresses were 0.3, 0.025, 0.008, and 0.005 Pa, respectively. It has been reported that shear stress ranges from 0.1 to 0.6 Pa in the venous system and from 1 to 15 Pa in the arterial system [45,46]. Therefore, this study focused on low shear stress conditions. This range of speeds is reflective of the typical movement of RBCs in capillaries, which generally varies between 0.1 and 1.0 mm/s, thereby effectively simulating the low-speed side. In the high-speed region of 10 mm/s, the transit time through the stenosis is as brief as 0.25 s. This necessitates enhancements in the performance of the imaging system. Implementing these improvements across a broad spectrum of conditions presents a future challenge. Furthermore, as shown in Figure 5B,C, this study focused on the initial RBC diameter *H*(0) and RBC height *H*(*t*) in the load application direction after *t* seconds of unloading. The Young’s modulus of RBCs varies based on the measurement technique employed, with reported values including 36 ± 7 Pa [47], 1.87 ± 0.90 kPa [48], and 3.6 ± 0.02 kPa [23]. In contrast, the Young’s modulus of PDMS is approximately 1.5 MPa [49]. Therefore, because PDMS has a Young’s modulus that is approximately 40,000–4000 times higher than that of RBCs, we concluded that a compressive load could be applied to RBCs at the constriction without deforming the PDMS.

**Figure 6:**
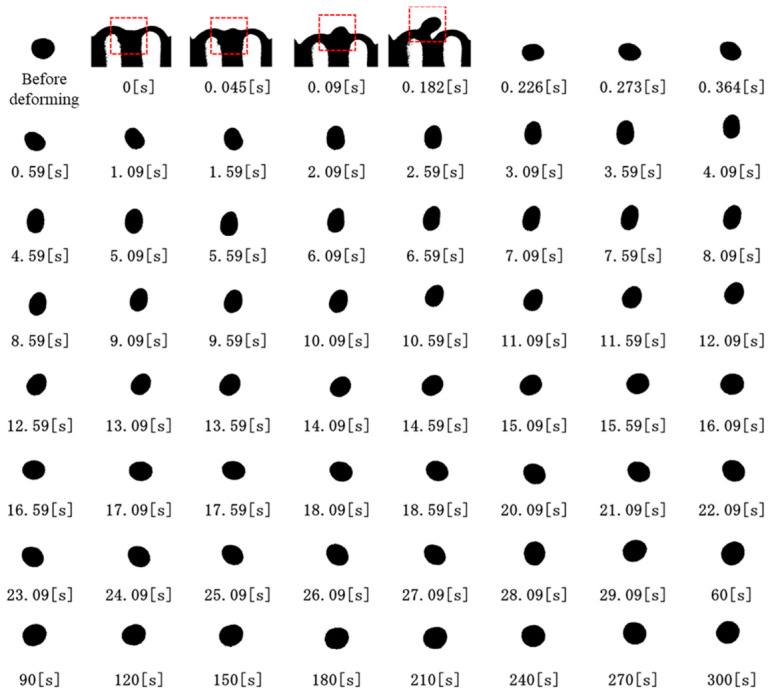
Example of a binary image of an RBC at a loading time of *T* = 300 s in a uniaxial compression experiment. In the duration immediately after release from the constriction, images were analysed at narrower time intervals. The area surrounded by the red dashed line represents RBCs that were released.

The image processing software “Image J” (version 1.54g, NIH, Bethesda, MD, USA) was employed to analyse the recovery behaviour. ImageJ was selected owing to its established reputation as a reliable analytical tool for cellular studies. Elapsed time was defined as the duration from the moment the RBCs were released from the constriction, with video recordings captured for up to 30 s following their release. Videos were taken for up to 30 s. Additionally, still images were captured every 30 s from 30 s to 300 s. Informed by previous research and preliminary experiments, the time intervals for analysis were refined to focus on periods where a high temporal resolution was particularly critical. The video was processed into still images frame-wise using the multimedia player ‘MPC-HC’. During the release of the RBCs from the constricted region, still images were captured at intervals of approximately 0.1 s. Following the complete release of the RBCs, images were taken at 0.5 s intervals for the first 20 s and at 1 s intervals from 20 to 30 s. An example binary image of an RBC at a loading time of *T* = 300 s in a uniaxial compression experiment is shown in Figure 6. The length of the minor axis of the RBC, approximated as an ellipse, was measured in the direction of the applied load, quantified by the number of pixels in that orientation.

## 3. Results

### 3.1. Manipulation Performance Results

**Figure 7:**
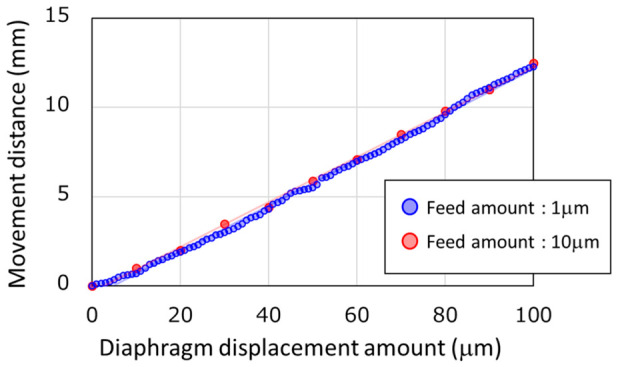
Relationship between diaphragm displacement and RBC movement distance.

Preliminary experiments assessing pump performance and design established that the movement distance of RBCs was 12.3 mm when the diaphragm was depressed by 100 µm. Furthermore, measurements obtained at 1 and 10 µm intervals of diaphragm depression revealed a linear relationship between the diaphragm’s displacement and the corresponding movement of the RBCs. Figure 7 shows the relationship between diaphragm displacement and RBC movement distance. The coefficient of determination was 0.998 for a feed amount of 10 µm and 0.997 for a feed amount of 1 µm. Since the movement distance was determined by the displacement regardless of feed amount, the RBC velocity could be determined from the movement of the piezo stage. In the current study, the diaphragm depth was adjusted to 50 µm for RBC manipulation, which was expected to result in different movement outcomes. Additionally, we aimed to elucidate the relationship between the feed amount and the movement of the piezo stage. The average displacements of RBCs for piezo stage displacements of 0.001, 0.01, 0.1, and 1 µm are listed in Table 1. The amount of movement was the average of ten trials. The RBCs could be positioned with a minimum resolution of 0.08 µm. The observed reduction in movement compared to the preliminary experiments can be attributed to modifications in the diaphragm’s shape and volume, specifically tailored for RBC manipulation. Additionally, as listed in Table 1, the travel distance tended to decrease when the feed command value was low. One potential explanation for this phenomenon is the interaction between the RBCs and the bottom of the channel. For example, the influence of gravity may have become more pronounced in the direction of the bottom surface owing to a decrease in the flow rate [50]. Moreover, the channels, constructed from PDMS, demonstrate hydrophobic properties and possess a negative charge in liquid environments, which can lead to the adsorption of proteins and cells. This interaction may have resulted in increased resistance when RBCs made contact with the channel’s bottom. The reported values pertain to manipulations conducted within a channel with a height of 3.6 µm and a width of 200 µm, excluding the constriction. The analysis validated a reduction in cross-section during constriction, accompanied by an average 7.4-fold increase in RBC migration speed. These findings facilitate the manipulation of RBCs within the constricted region.

### 3.2. Uniaxial Compression Experiment

**Figure 8:**
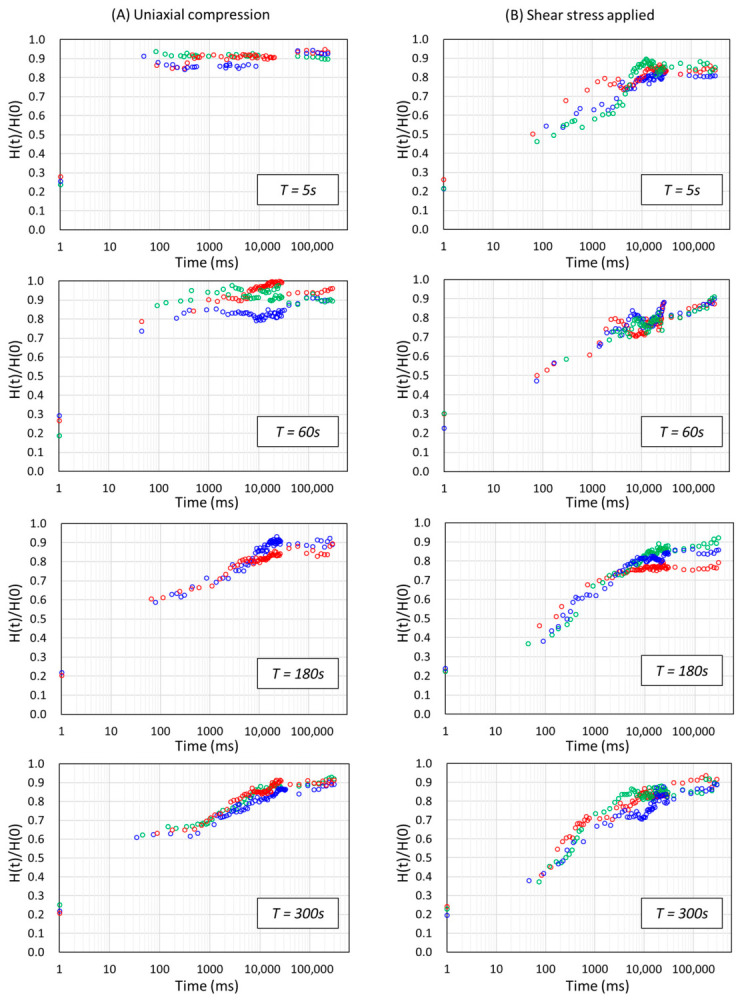
Correlation between elapsed time after unloading and shape recovery rate. (**A**) Uniaxial compression experiment; (**B**) shear stress experiment. The shape recovery rate versus elapsed time at *T* = 5, 60, 180, and 300 s is shown. *H*(*t*)/*H*(0) was calculated as the RBC shape recovery rate. The horizontal and vertical axes represent the elapsed time on a logarithmic scale and shape recovery rate of RBCs, respectively. Different colours indicate independent experimental trials conducted under the same conditions. In most cases, three independent experiments were performed (n = 3), whereas one condition included two independent experiments (n = 2).

Uniaxial compression experiments were conducted following the methodology outlined in the “Experimental Method” section and shown in Figure 5. By analysing the captured shape recovery behaviour, we obtained the diameter dimension *H*(*t*) of the RBCs in the loading direction over time. The ratio of these data to the dimensions before deformation, *H*(0), *H*(*t*)/*H*(0), was calculated as the shape recovery rate of the RBCs. The shape recovery rate versus elapsed time at *T* = 5, 60, 180, and 300 s is shown in Figure 8A. The experiment was conducted twice for *T* = 180 s and three times for *T* = 5, 60, and 300 s, with distinct colours assigned to each trial in the figure. To represent the elapsed time on a logarithmic scale, we marked the moment at which the RBCs were released from the stenosis as 1 ms. The horizontal axis denotes the elapsed time on a logarithmic scale, whereas the vertical axis represents the shape recovery rate of the RBCs. For all loading durations, the interval between the data points was 1 ms and the subsequent data points ranged from 30 to 50 ms, a limitation attributed to the camera’s frame rate. The maximum frame rate of BU1203MCF was 30 fps; hence, a difference of 0.033 was observed between the first and second frames. The elastic shape recovery decreased with increasing *T*. Notably, a distinct overshoot in recovery was observed at the onset of the recovery phase, specifically at *T* = 5 s. Additionally, complete recovery was not achieved under any of the tested conditions, validating the occurrence of plastic deformation.

### 3.3. Shear Stress Experiment

Similar to the analysis presented in the “Uniaxial Compression Experiment” section, which focused on uniaxial compression experiments, we also investigated the recovery behaviour of RBCs under shear stress conditions. The shape recovery rate versus elapsed time at *T* = 5, 60, 180, and 300 s is shown in Figure 8B. Each experimental condition was replicated three times, with distinct colours assigned to the plots for clarity. The experimental results shown in Figure 8A,B were obtained from the same donor, rather than from pooled samples collected from multiple subjects. This approach was adopted to minimise inter-subject variability and ensure consistency in the measurement results. To represent the elapsed time on a logarithmic scale, we marked the moment at which the RBCs were released from the stenosis as 1 ms. Across all experimental trials, the recovery rate observed between 100 and 1000 ms was notably lower compared with the results shown in Figure 8A, which pertains to the uniaxial compression experiment.

## 4. Discussion

**Figure 9:**
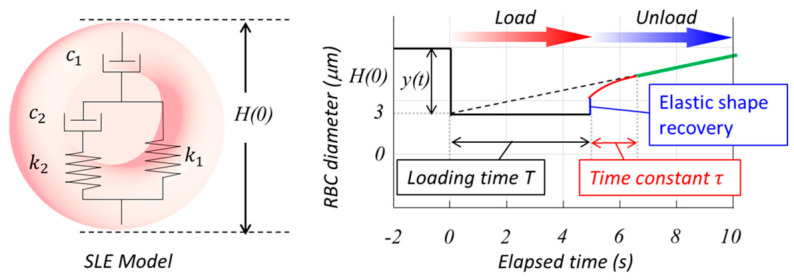
SLE model of RBCs and a schematic of the diameter change process when the loading time *T* = 5 s. For the change process, the time and diameter are represented on the horizontal and vertical axes, respectively.

The application of shear stress resulted in a tendency for shape recovery that differed from that observed in uniaxial compression experiments. Furthermore, to quantitatively evaluate the shape recovery, we calculated the characteristic recovery time constant, which represents the duration required to achieve an equilibrium state. To derive the characteristic recovery time constant τ from the results obtained in the “Results” section, we employed the SLE model formula introduced in the “Introduction” section. SLE models RBCs as viscoelastic bodies, utilising a straightforward framework that integrates spring and viscosity constants. This enables the SLE model to effectively simulate the response of RBCs to external forces [51]. Its practicality lies in its ability to encapsulate complex viscoelastic phenomena with a minimal number of parameters. Furthermore, the SLE model has a proven track record in analysing the viscoelastic behaviour of RBCs under uniaxial stress conditions [30], rendering it an appropriate framework for this study. Figure 9 illustrates the SLE model of RBCs and the diameter change process at a loading time of *T* = 5 s.

As indicated schematically by the blue line in Figure 9, RBCs exhibited rapid elastic shape recovery within approximately 0.1 s, followed by slower viscous shape recovery over approximately 10 s. The red line in the figure represents the viscous shape recovery, which unfolds over several seconds, with the time taken to reach the equilibrium state being designated as the characteristic recovery time constant. The viscoelastic properties of RBCs within the SLE model can be represented by two spring constants ($k_{1}$, $k_{2}$) and two viscous constants ($c_{1}$, $c_{2}$). The shape recovery process in the SLE model is expressed as follows:

(1) $$\frac{y(t)}{F}=\frac{H\left(0\right)-H(t)}{F}=\frac{1}{k_{1}}\left(1-\frac{k_{2}}{k_{1}+k_{2}}\cdot e^{-t/\tau }\right)+\frac{t}{c_{1}}$$

where y(t) represents the difference between the diameter before compression and the thickness of the RBCs during shape recovery. The time–diameter correlation data given in the “Results” section were incorporated into the aforementioned SLE model. Furthermore, F represents the load, with the extent of shape recovery being directly proportional to this force. If the initial diameter was 8 µm and compression reduced it to 3 µm, with a spring constant of 5.0 × 10^−6^ N/m, the resulting force was 25 pN. Therefore, although this served only as reference data, the force was estimated to be on the order of several tens of pN. As indicated by the blue line in Figure 9, the shape recovery behaviour of RBCs demonstrated a rapid elastic recovery phase that occurred within a brief timeframe of approximately 0.1 s, followed by a slower viscous recovery phase that spanned approximately 10 s. The viscous shape recovery following elastic shape recovery could be divided into two stages. The first stage, characterised by viscous shape recovery that occurred over several seconds, corresponded to the segment indicated by the red line in Figure 9. The duration of this initial stage of viscoelastic recovery could be quantified by the characteristic recovery time constant τ, expressed as follows:

(2) $$\tau =\frac{c_{2}\left(k_{1}+k_{2}\right)}{k_{1}k_{2}}\;$$

The second stage, viscous shape recovery, was a more gradual process that followed the first stage and was influenced by the duration of the compressive load. This phase is represented by the green line in Figure 9. The rate of elastic shape recovery observed in the brief timeframe (approximately 0.1 s) decreased as *T* increased (Figure 8). This trend is consistent with previous studies [30]. Specifically, $c_{2}$ changed in a time-dependent manner, increasing by more than tenfold after 180 s. Conversely, in the shear stress application experiments, the rate of elastic shape recovery remained consistent regardless of increases in *T*. When *T* = 300 s, the observed speed was 0.008 mm/s, accompanied by a correspondingly low shear stress (0.005 Pa). However, when compared with *T* = 300 s in the uniaxial compression experiment, the suboptimal elastic shape recovery rate was evident. It can be observed that the two spring constants ($k_{1}$, $k_{2}$) decreased and the two viscous constants ($c_{1}$, $c_{2}$) increased, although the reason for this remains unclear. If the cell membrane is regarded as a collection of springs [52], it is possible that some of these springs were damaged, causing the values of k to decrease. Alternatively, dissociation and recombination of the spectrin network, one of the cytoskeletal components, may have contributed to the increase in c [53]. It is also possible that ATP, the cellular energy source, decreased due to shear stress loading, thereby affecting c [54]. Another phenomenon unique to the shear stress experiments was a delay in recovery following the elastic jump (exponential relaxation). This may reflect a change in $c_{2}$. There has been little systematic research into the mechanisms underlying phenomena occurring over timescales slower than 100 ms. However, there are reports that RBCs attempt to recover their original shape following damage (τ > 4 h) [55]. These observations may indicate partial membrane damage or alterations in cytoskeletal mechanics induced by shear loading. This observation suggests that shear stress may significantly influence the elastic shape recovery rate of RBCs. To further elucidate this relationship, we express the diameter of the RBCs, H(t), using Equation (1).

(3) $$H\left(t\right)=H(0)+y\left(t\right)=H(0)+\frac{F}{k_{1}}+\frac{t}{c_{1}}-\frac{F}{k_{1}}\left(-\frac{k_{2}}{k_{1}+k_{2}}\right)e^{-t/\tau }$$

Furthermore, using constants $A_{1}$ and $A_{2}$ in Equation (3), H(t) can be expressed as follows:

(4) $$H(t)=A_{1}-A_{2}\cdot e^{-t/\tau },$$

(5) $$\left\{\begin{array}{c} A_{1}=H(0)+\frac{F}{k_{1}}+\frac{t}{c_{1}}\\ A_{2}=\frac{F}{k_{1}}\left(-\frac{k_{2}}{k_{1}+k_{2}}\right) \end{array}\right.$$

**Figure 10:**
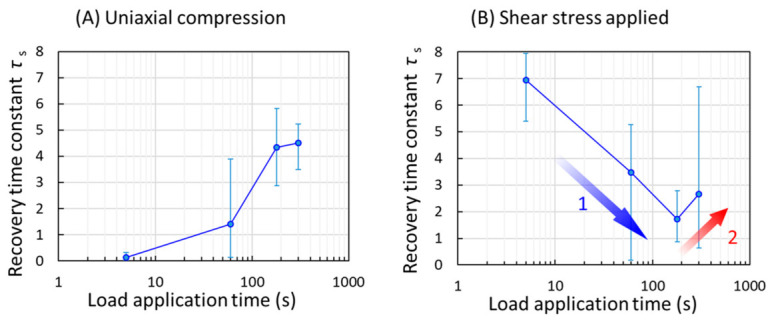
Correlation between load application time and characteristic recovery time constant. (**A**) Uniaxial compression experiment; (**B**) shear stress experiment. The horizontal axis represents the applied load time on a logarithmic scale, whereas the vertical axis shows the red recovery time constant until the equilibrium state was reached.

In Equation (4), τ was determined by fitting the experimental data through the least squares method, utilising parameters $A_{1}$ and $A_{2}$. The Excel Solver function was used for fitting. “GRG nonlinear” was utilised as the ‘solution method’ of the solver. The analysis results were summarised as the shape recovery rate H(t)/H(0). The τ obtained under uniaxial compressive stress is shown in Figure 10A. τ increased with increasing load time *T*. Specifically, an increase in τ signified a longer duration required for shape recovery. Notably, τ demonstrated a rapid increase at *T* = 180 s, with no significant difference observed between *T* = 180 s and 300 s. This tendency is similar to the behaviour of τ in previous research [30]. The results for v obtained when shear stress was applied are shown in Figure 10B. Here, τ decreased as T increased up to 180 s, followed by an increase at *T* = 300 s. This trend differed from that observed in the uniaxial compression experiment, suggesting that shear stress, in combination with physical contact with the channel wall, may have influenced RBC recovery behaviour. It should also be noted that RBCs in the present system were subjected not only to fluid shear stress but also to geometric confinement and physical contact with the channel wall within the narrow constriction (3.6 × 3.0 µm cross-section). The estimated compressive stress acting on RBCs inside the constriction was approximately 25 Pa, which was substantially greater than the applied fluid shear stress (maximum approximately 0.3 Pa). Therefore, the observed deformation and recovery behaviour were considered to be governed predominantly by constriction-induced mechanical loading, including compression and confinement effects, rather than by shear stress alone. At the same time, deformation during passage through the constriction under the present low shear stress conditions was likely limited. However, compared with compression-only loading, differences in post-loading RBC recovery behaviour were observed when shear stress was additionally applied, even at relatively low levels. Although statistically significant differences were not consistently observed across all loading conditions, particularly at *T* = 60, 180, and 300 s, a tendency toward altered recovery behaviour was observed. These findings suggest that even a relatively small shear component may influence RBC recovery dynamics following constriction. In particular, the *p*-value for τ at *T* = 5 s was 0.0016, indicating a statistically significant difference under this condition. As shown in Equation (2), $c_{2}$ contributed to the increase in the recovery time constant. Previous studies have reported that $c_{2}$ increases after loading durations exceeding 180 s due to prolonged compression by the channel wall [30]. The present findings may suggest that shear loading also contributes to changes in recovery behaviour, although the precise mechanism remains unclear. Shear loading may induce alterations in membrane or cytoskeletal mechanics. For example, changes in membrane structure [53], spectrin network dynamics [54], or ATP-dependent viscoelastic regulation [55] may contribute to altered recovery behaviour. However, these interpretations remain speculative and require further experimental validation. At *T* = 5 s, the average moving speed of RBCs was 0.5 mm/s under the present experimental conditions. In contrast, at *T* = 180 s, the average speed decreased to 0.014 mm/s, corresponding to approximately 1/36 of that at *T* = 5 s, with the associated shear stress decreasing from 0.3 Pa to 0.008 Pa. This reduction in velocity may explain the trend shown as “1” in Figure 10, suggesting a reduced contribution of shear loading at lower velocities. Furthermore, at *T* = 300 s, the average moving speed decreased further to 0.008 mm/s, approaching conditions similar to those used in uniaxial compression viscoelasticity measurements. Therefore, the trend shown as “2” in Figure 10B may be influenced more strongly by prolonged loading duration than by shear stress. Nevertheless, even under extremely low shear stress conditions (0.005 Pa; flow velocity: 0.008 mm/s), a reduction in the elastic jump was observed, suggesting that shear loading may still contribute to recovery behaviour. Because both loading duration and shear stress may affect the response, clear statistical differences were not consistently observed at *T* = 60, 180, or 300 s.

Several studies have investigated cell manipulation and sorting using microfluidic devices. However, relatively limited attention has been paid to the potential effects of physical contact with channel walls or atypical flow conditions on cellular behaviour and recovery characteristics. The present findings suggest that not only loading duration but also flow velocity may influence post-loading RBC recovery. Although statistically significant differences were observed primarily at *T* = 5 s, tendencies toward altered recovery were observed across multiple loading conditions. From an application perspective, particularly in RBC sorting and manipulation, reducing loading duration and avoiding unnecessarily high flow velocities may help minimise potential alterations in recovery behaviour. However, because the present study analysed a limited number of RBCs (n = 3 for each condition), additional experiments with larger sample sizes will be required to further validate the reproducibility and statistical robustness of these findings and support the development of practical design guidelines. One possible factor contributing to delayed recovery is loading-induced alterations in membrane or cytoskeletal mechanics. However, the precise mechanism remains unclear. Future studies should investigate cytoskeletal dynamics during loading and recovery. Because cytoskeletal structures can be visualised using fluorescence imaging, real-time observation combined with accurate RBC tracking may help clarify the underlying mechanism. These future developments may contribute to a better understanding of how combined mechanical loading influences RBC recovery behaviour in confined microenvironments.

## 5. Conclusions

This study specifically examined the effects of physical contact between the channel walls and cells. First, we developed a microfluidic device integrated with a pump that completely halted cells within a constriction, applying a load while enabling flow at variable speeds. Uniaxial compression viscoelasticity measurements were performed on RBCs, and the recovery behaviour of these cells was observed and evaluated, validating their consistent shape recovery behaviour with that reported in previous studies. Therefore, we can assert that our device possesses adequate specifications for analysing cell behaviour, particularly when compared with conventional manipulation systems that rely on feedback control mechanisms using a high-speed camera. As a novel advancement, we established technology capable of processing channels up to 2500 µm in length on a 3.6 × 3.0 µm cross-section. Consequently, RBCs could be manipulated at speeds ranging from 0.008 to 0.5 mm/s, enabling experiments that allow investigation of the effects of shear stress. The shape-recovery behaviour differed from that of the uniaxial compression experiment, with complete stoppage within the constriction. Notably, the recovery time constant demonstrated up to a 30-fold variation depending on the conditions: when a load was applied at a complete stop for 5 s versus when it was applied while flowing at a rate of 0.5 mm/s for the same duration. Additionally, our findings indicated that the recovery rate diminished between 100 and 1000 ms post-unloading, in contrast to the results from the uniaxial compression experiment. This suggests that the interplay between ‘flow velocity’ and ‘loading time’ may influence the deformation behaviour of cells. However, the current experimental setup does not facilitate investigations across a broad spectrum of conditions. To address this limitation, we aim to develop flow channels and manipulation techniques that are better suited for exploring the effects of these variables on cell damage. Notably, previous studies have not adequately addressed the potential for cellular damage or morphological changes caused by channel walls or irregular flow patterns. We anticipate that the insights gained from this research will contribute to the design of flow channels that minimise cellular damage.

## Figures and Tables

**Table 1:** Relationship between piezo command value and movement amount.

Piezo Stage Feed Amount [µm]	Average Movement Amount. [µm]
0.001	0.08
0.01	0.79
0.1	9.1
1	92.9

## Data Availability

The original contributions presented in this study are included in the article. Further inquiries can be directed to the author.

## Abbreviations

The following abbreviations are used in this manuscript:

RBCs	Red blood cells
SLE	Standard linear elasticity
PDMS	Polydimethylsiloxane
PGMEA	Propylene glycol monomethylether 1-methoxyl-2-propanol
